# Comparison of rigid and deformable registration through the respiratory phases of four-dimensional computed tomography image data sets for radiotherapy after breast-conserving surgery

**DOI:** 10.1097/MD.0000000000009143

**Published:** 2017-12-15

**Authors:** Aiping Zhang, Jianbin Li, Heng Qiu, Wei Wang, Yanluan Guo

**Affiliations:** a School of Medicine and Life Sciences, University of Jinan-Shandong Academy of Medical Sciences; b Department of Radiation Oncology; c Breast Cancer Center, Shandong Cancer Hospital Affiliated to Shandong University, Jinan, Shandong Province; d The Third Hospital of Jinan, China.

**Keywords:** breast-conserving surgery, deformable image registration, four-dimensional computed tomography, respiratory movement, rigid image registration

## Abstract

**Background:**

The aim of this study was to compare the geometric differences in gross tumor volume (GTV) and surgical clips propagated by rigid image registration (RIR) and deformable image registration (DIR) using a four-dimensional computed tomography (4DCT) image data set for patients treated with boost irradiation or accelerated partial breast irradiation after breast-conserving surgery (BCS).

**Methods:**

The 4DCT data sets of 44 patients who had undergone BCS were acquired. GTV and selected clips were manually delineated on end-inhalation phase (CT_0_) and end-exhalation phase (CT_50_) images of 4DCT data sets. Subsequently, the GTV and selected clips from CT_0_ images were transformed and propagated to CT_50_ images using RIR and DIR, respectively. The geometric differences in GTV and surgical clips from DIR were compared with those of RIR.

**Results:**

The mean Dice similarity coefficient (DSC) index was 0.860 ± 0.042 for RIR and 0.870 ± 0.040 for DIR for GTV (*P = *.000). The three-dimensional distance to the center of mass (COM) of the GTV from RIR was longer than that from DIR (1.22 mm and 1.10 mm, respectively, *P = *.000). Moreover, in the anterior–posterior direction, displacements from RIR were significantly greater than those from DIR for both GTV (0.70 mm and 0.50 mm, respectively) and selected clips (upper clip, 0.45 mm vs 0.20 mm; inner clip, 0.55 mm vs 0.30 mm; outer clip, 0.40 mm vs 0.20 mm; lower clip, 0.50 mm vs 0.25 mm) (*P = *.000). However, in the left–right and superior–inferior directions, there were no significant displacement differences between RIR and DIR for GTV and the selected clips (all *P* > .050).

**Conclusion:**

DIR can improve the overlap for GTV registration from CT_0_ to CT_50_ images from 4DCT scanning. Furthermore, DIR is superior to RIR in reflecting the displacement of GTV and selected clips in the anterior–posterior direction induced by respiratory movement.

## 1 Introduction

Breast-conserving therapy (BCT) is the standard of care for early stage breast cancer. Accurately defining the tumor bed (TB) volume for radiation treatment planning is crucial for BCT to ensure proper coverage of tumors and spare organs at risk.^[1–3]^ The critical factors leading to uncertainties in TB position between treatment and planning computed tomography scans during BCT include setup errors, respiration-induced target movements, and breast deformation. During radiotherapy, treatment and planning computed tomography scans are performed while the patient breathes freely, an activity subject to inherent motion artifacts.^[4]^ Therefore, target motion caused by respiration during free breathing has recently become a focus of radiotherapy research.^[5]^ By synchronizing computed tomography image acquisition to respiratory curves, four-dimensional computed tomography (4DCT) is used to assess respiratory-induced target motion and to determine internal target volumes for BCT.^[6,7]^

Although 4DCT images provide details regarding how the delineation of gross tumor volume (GTV) is influenced by respiratory motion, manually delineating GTVs at each phase of 4DCT scans is time consuming and labor intensive. The development of image registration has enabled advances in image-guided radiotherapy. Rigid image registration (RIR), which is widely used in many cancer centers, offers increased efficiency by aligning one CT image with another to accurately define the GTV for treatment.^[8,9]^ However, RIR is also subject to inaccuracies caused by rigidly registering a nonrigid tissue and may not account for changes in the weight of the patient between scans, changes in the positioning of the patient, and soft-tissue displacements due to breathing. By tracking voxel-to-voxel changes from one CT image to another, deformable image registration (DIR) can correct for these changes by mapping between volume elements in one image and the corresponding volume elements in a subsequent image.^[10]^ Previous studies relying on qualitative evaluations have suggested that DIR is almost always more accurate than RIR for assessing lung cancer.^[11,12]^

Although many studies investigating the usefulness of DIR have been conducted, its clinical impact on defining target volumes in 4DCT scans for treating breast cancer has not been reported. Therefore, the objective of this study was to evaluate the efficacy of DIR in assessing respiratory movements during intrafraction irradiation compared with the efficacy of RIR in 4DCT image data sets of patients undergoing radiotherapy after breast-conserving surgery (BCS). The results provide reference data for the application of 4DCT scans and image registration techniques to improve the delineation of target volumes influenced by respiratory motion.

## 2 Materials and methods

### 2.1 Patients

The study included 44 female patients with early stage breast cancer who had undergone BCS in our department between November 2014 and August 2016. The patients’ characteristics are listed in Table 1. The average interval from surgery to radiotherapy was 12 weeks (range, 2–24). Of the 44 patients, 68% were diagnosed with hormone receptor-positive breast cancer; 91% of these patients were receiving concurrent hormone therapy plus radiotherapy after BCS, and the other 9% were receiving sequential radiotherapy and hormone therapy after BCS. To improve delineation accuracy and consistency, all enrolled patients had 5 or more surgical clips fixed to the central bottom and lateral edges of the excision cavity to mark the lumpectomy cavity (LC) boundaries. Patients with restricted arm movement after surgery and poor pulmonary function were excluded. This study was approved by the institutional research ethics board of Shandong Cancer Hospital. Written informed consent was obtained from all patients.

**Table 1:**
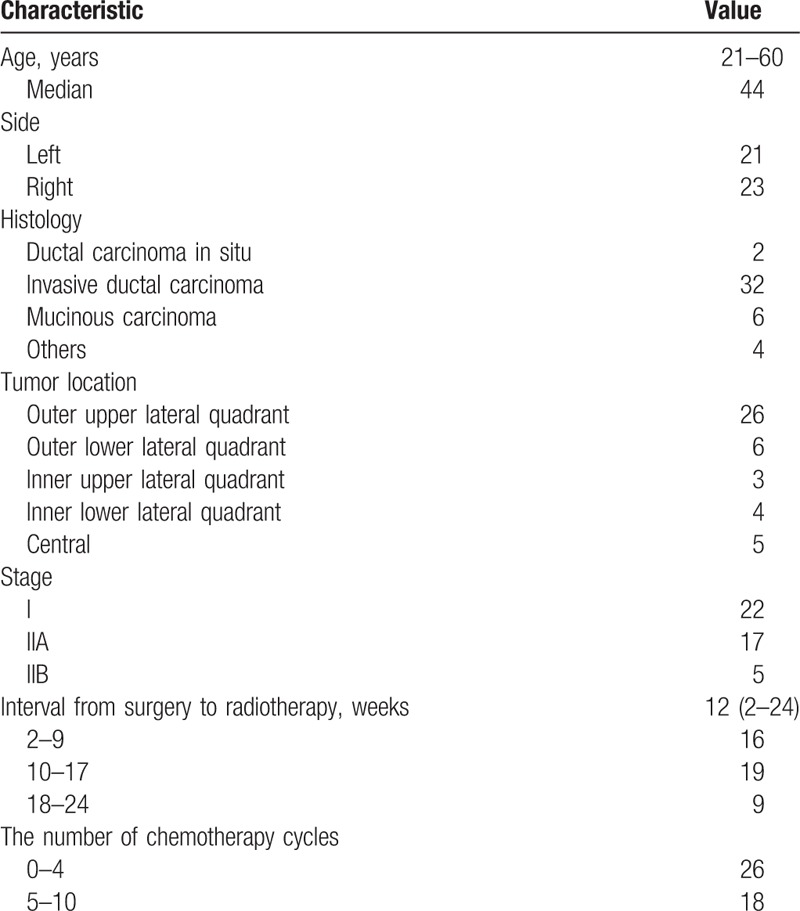
Patient and tumor characteristics.

### 2.2 4DCT scans and image acquisition

All 44 patients were immobilized in the supine position on a breast board with both upper limbs outreached and lifted on arm brackets. During free breathing, 4DCT images were acquired, with a thickness of 3 mm, at the conclusion of a standard CT simulation using a 16-slice Brilliance Big Bore CT scanner (Philips Medical Systems, Inc., Cleveland, OH). Respiratory signals were recorded with a Varian Real-time Positioning Management (RPM) gating system (Varian Medical Systems, Palo Alto, CA) by tracking the trajectory of infrared markers placed on the patient's abdomen. GE Advantage 4D software (GE Healthcare, Waukesha, WI) sorted the reconstructed the 4DCT images into 10 respiratory phases based on these tags, with 0% corresponding to the end-inhalation phase (CT_0_) and 50% corresponding to end-exhalation phase (CT_50_). Next, the constructed 4DCT images were transferred to MIMvista version 6.1.0 (MIM Software, Cleveland, OH) for structure delineation.

### 2.3 Manual contouring of GTVs and selected clips

GTVs were manually contoured by the same radiation oncologist based on the placement of the surgical clips as a guideline on the CT_0_ and CT_50_ images of the 4DCT data sets, using seroma as a reference. GTVs delineated on the CT_0_ and CT_50_ images were defined as GTV_0_ and GTV_50_, respectively. The surgical clips representing the superior, inferior, posterior, and lateral boundaries of the LC were selected and marked as the upper, lower, inner and outer clips, respectively.

### 2.4 Registration procedure

For RIR and DIR, both the manually contoured GTV and selected clips on the CT_0_ image were propagated to the CT_50_ image using the MIM Registration package. The registration methods were largely automated, with user interaction limited to defining the region of interest for registration. The DIR process starts with a rigid registration of the CT_0_ images to the CT_50_ images. The user performs an automatic rigid registration and then evaluates the rigid registration. GTV_0_ was named 
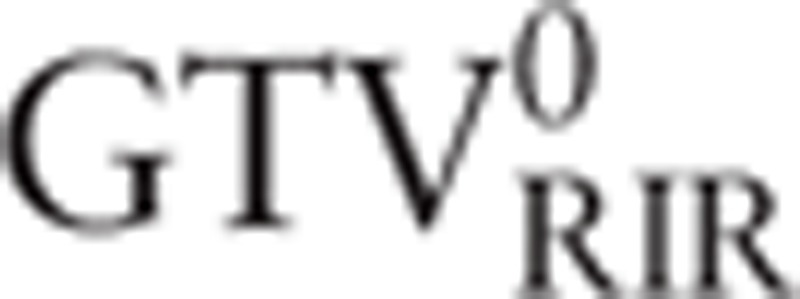
 after RIR. Once the rigid registration was accepted, the deformable registration and adaptive contouring module in the MIM software deformed the CT_0_ images to match the CT_50_ images. Eventually, the software, based on the calculated deformation matrix, mapped the CT_0_ contours (GTV and the selected clips) onto the CT_50_ images. GTV_0_ was named 
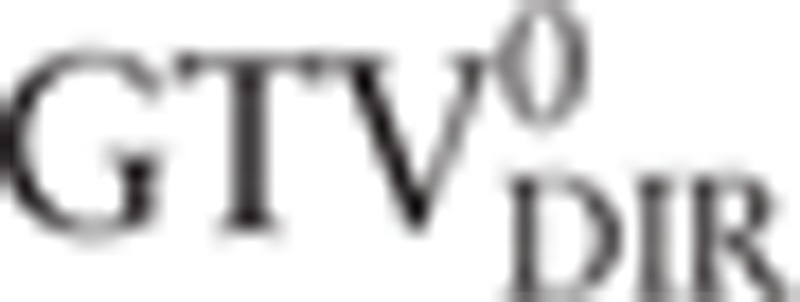
 after deformation via DIR.

### 2.5 Three-dimensional coordinate measurement

The MIM software automatically outputs the 3D coordinates of the 2 sets of registered images for both the geometric center of the GTV and the selected clips, and these coordinates were recorded. Next, the peak-to-peak displacement (the maximum value of the coordinate minus the minimum value of the coordinate) of the clips and the geometric center of the GTV were obtained and marked as Δ*x*, Δ*y*, and Δ*z*. The 3D distance to the center of mass (COM) was calculated as follows: 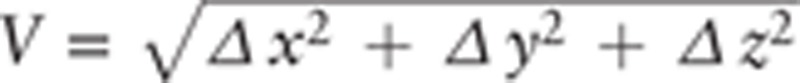. The results for the GTV and selected clips from DIR were compared with those from RIR.

### 2.6 Statistical analysis

To determine the degree of overlap between GTVs obtained using RIR and DIR, we used the Dice similarity coefficient (DSC). The DSC is a commonly used metric in medical imaging and contouring studies^[26,27]^ and is defined as follows: DSC (*A*, *B*) = 2|*A*∩*B*|/(|*A*| + |*B*|). This metric has values ranging from 0, for no overlap, to 1, for perfect agreement between volumes.

Statistical significance was measured using paired *t*-tests if the Shapiro–Wilk normality test was passed (*P* > .05); otherwise, Wilcoxon signed-rank tests were used. Analyses were performed in SPSS 17.0. Results were considered statistically significant at *P* < .05.

## 3 Results

### 3.1 Comparisons of GTV and DSC between RIR and DIR

**Figure 1:**
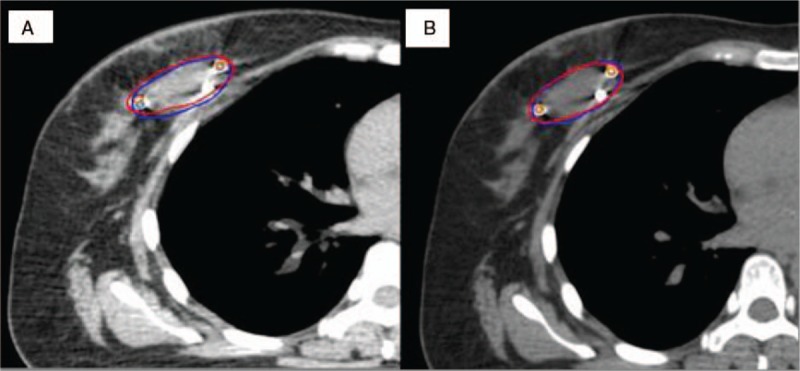
Original manual contour of the gross tumor volume at CT_50_ (blue) and the registered gross tumor volume at CT_0_ (red) using (A) rigid image registration and (B) deformable image registration. CT = computed tomography^^.

**Figure 2:**
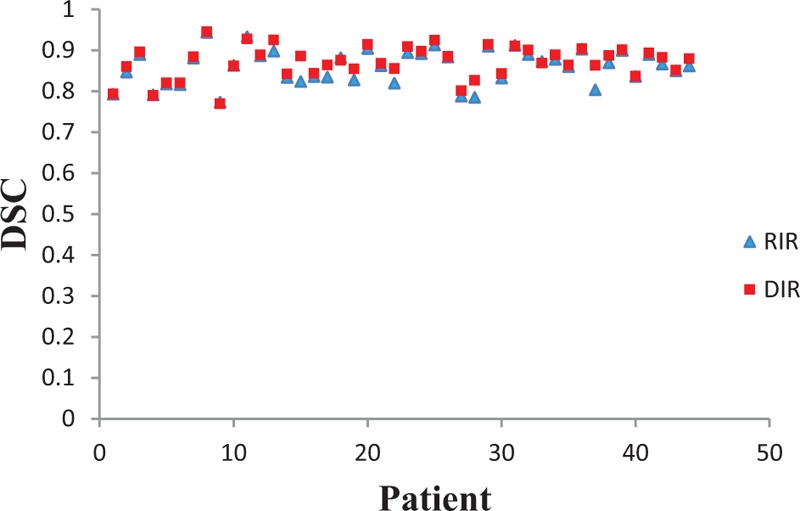
Dice similarity coefficient (DSC) index values for the rigid image registration (RIR) and deformable image registration (DIR) methods for individual patients. DIR = deformable image registration, DSC = Dice similarity coefficient,

The image registration results are shown in Figure 1. GTV_0_, GTV_50_, 
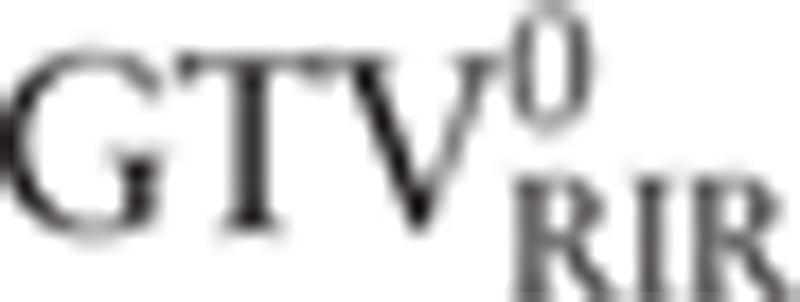
 and 
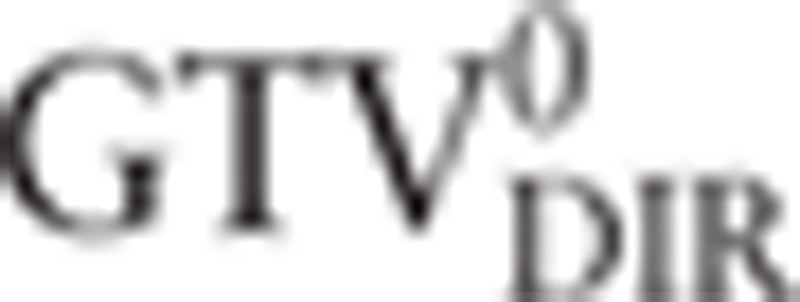
 are listed in Table 2. There were no significant differences between GTV_0_ and GTV_50_ or between GTV_50_ and 
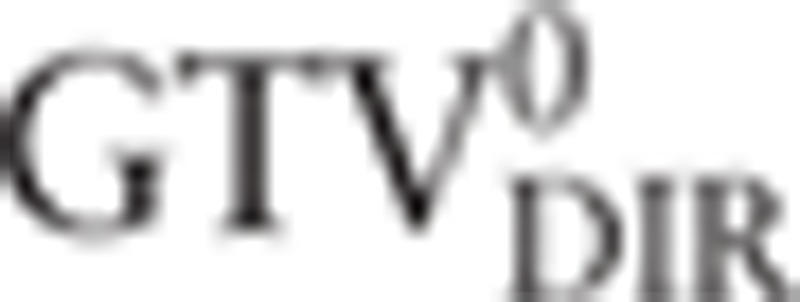
 (all *P* > .050). Similarly, no significant difference was observed between GTV_50_–
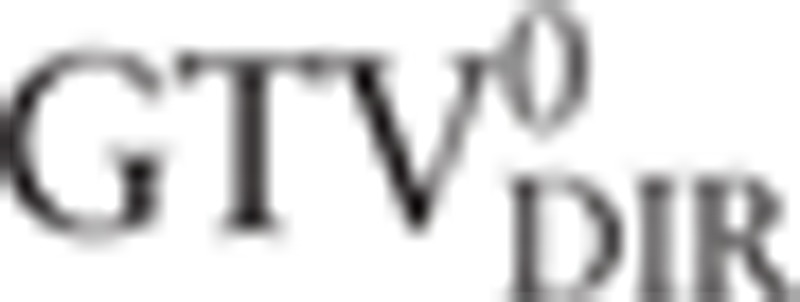
 and GTV_50_–
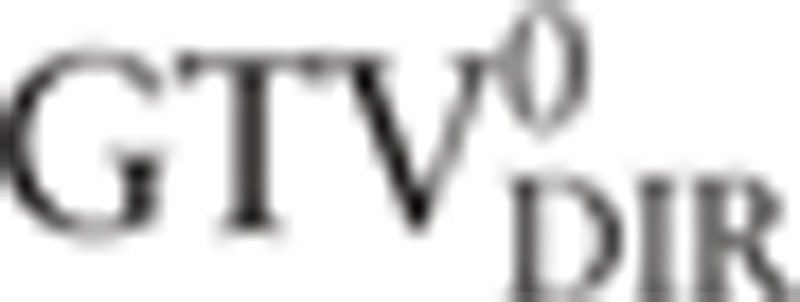
 (*z* = −1.64, *P = *.101). The average Dice similarity coefficient (DSC) was 0.86 (95% confidence interval: 0.85–0.87) for RIR and 0.87 (5% confidence interval: 0.86–0.88) for DIR (Fig. 2). The mean DSC for DIR was significantly higher than for RIR (*P = *.000).

**Table 2:**
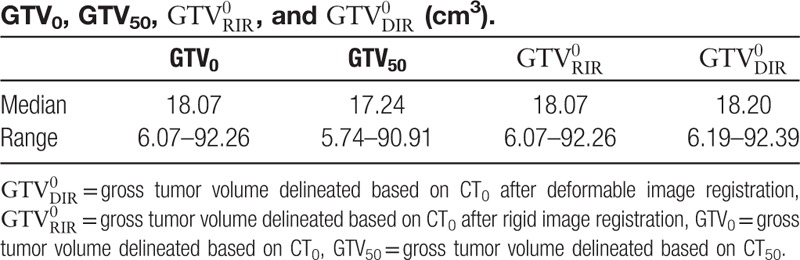


### 3.2 Three-dimensional comparisons of displacement

The centroid displacement of both GTVs and selected clips in the left–right, anterior–posterior and superior–inferior directions and the three-dimensional (3D) distances from the center of mass (COM) are listed in Table 3. There were no significant displacement differences between RIR and DIR in the left–right and superior–inferior directions for GTVs and the four selected clips (*P* > .05). However, in the anterior–posterior direction, displacements from RIR were significantly greater than those from DIR (*P = *.000). 3D distances to the COM from RIR showed greater movement than those from DIR (*P = *.000).

**Table 3:**
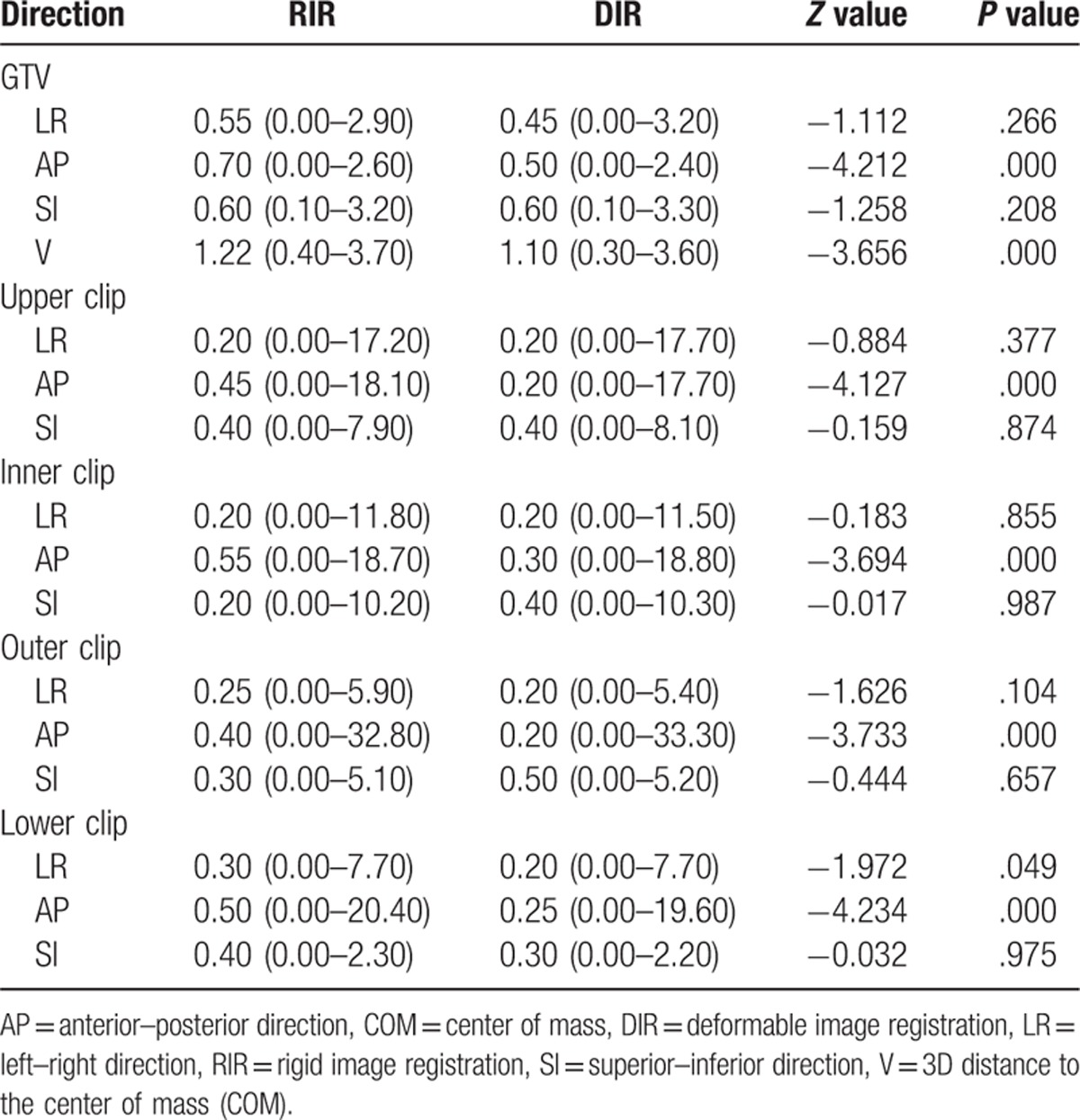
Centroid displacement of GTVs and selected clips from RIR and DIR (mm).

## 4 Discussion

Intra- and, in particular, interobserver variation is an important issue during the delineation of the TB on CT scans when performing BCT. A standard contouring protocol can be used to decrease intra- and interobserver variability when delineating the TB volume.^[13,14]^ Therefore, in our study, all delineations were performed by the same radiation oncologist according to unified guidelines to decrease geometric uncertainties. GTVs differed by as much as 6.5% between 
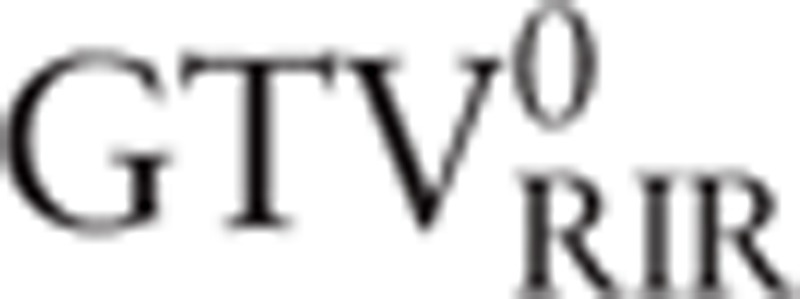
 and GTV_50_ and 5.8% between 
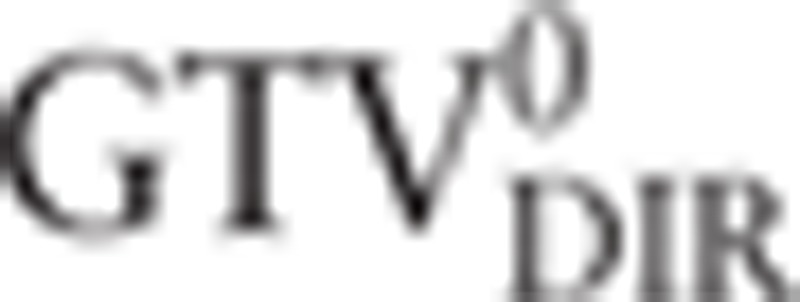
 and GTV_50_. Moreover, there was no significant difference in volume variation between RIR and DIR. GTV variation was similar for both registrations, suggesting that no sizeable volume progression occurred during DIR. In addition to comparing variations in target volume, we also analyzed differences in DSC values to evaluate volume alignment. A significant improvement was observed in the DSC for registration between 
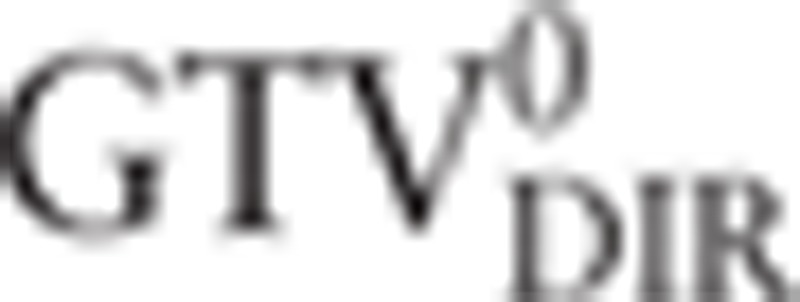
 and GTV_50_ compared with that between 
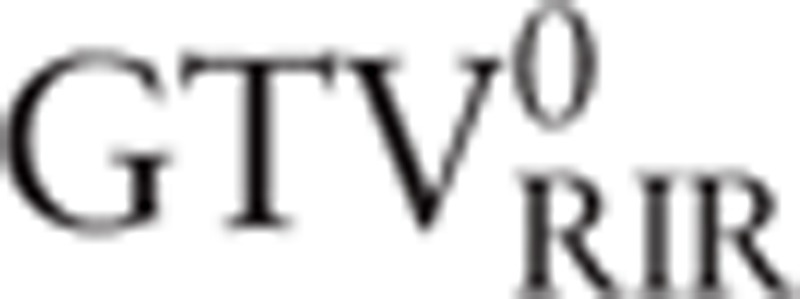
 and GTV_50_ (Fig. 2). This can be explained by the fact that RIR allows a linear or uniform transformation of all voxels in the image set within six degrees of freedom. This means it has its own associated inaccuracies in registering nonrigid tissues. In contrast, DIR is able to compute nonlinear and nonuniform relationships between volume elements across imaging datasets. For this reason, it is increasingly applied in radiation oncology to register image sets. Other authors have also reported that DIR is superior to RIR with respect to variations in target volume and shape. For example, Fortin et al^[15]^ observed that GTVs differed by as much as 30% between RIR and DIR for ten head and neck cancer patients.

For the selected clips, as rigid structures, no significant differences between RIR and DIR were observed in displacements in the left–right and superior–inferior directions when registering from CT_0_ to CT_50_ (*P* > .05). However, significant differences in displacements in the anterior–posterior direction were observed in the similar comparison (*P = *.000). Clips implanted at the boundaries of the surgical cavity are commonly used to delineate the TB volume and measure displacement for accelerated partial breast irradiation (APBI).^[16]^ Wang et al^[17]^ measured clip displacement and the geometric center of all clips based on 4DCT during free breathing and observed similar trends for the clips and their geometric center. Analogous to results from previous studies, our comparison of GTV displacement from RIR and DIR in the left–right, anterior–posterior and superior–inferior directions showed results similar to those for the selected clips. This may be attributable to (1) the slice-by-slice approach use in DIR, which results in no significant deformation in the longitudinal location; (2) the fixed position of the patient, such as lifting and outreaching both hands and lying in a supine position on the breast brackets; (3) breast size and shape; and (4) the location of the surgical cavity.

For both TB boost and partial breast irradiation, it is important to consider 3D displacement difference in determining the internal target volume (ITV).^[18]^ We previously investigated respiratory-induced displacements in GTV.^[17,19]^ Wang et al^[17]^ measured geometric center displacement based on the 10 phases of 4DCT data sets during free breathing and determined that geometric center displacements in the left–right, anterior–posterior, and superior–inferior directions averaged 1.3 ± 0.4 mm, 2.0 ± 1.0 mm, and 1.9 ± 1.0 mm, respectively. Similarly, in our study, we concluded that centroid displacements between GTV_0_ and GTV_50_ in the left–right, anterior–posterior, and superior–inferior directions were 0.55 (0.00–2.90) mm 0.70 (0.00–2.60) mm, and 0.60 (0.10–3.20) mm, respectively. Despite these reports, it is unclear if the different image registration techniques provide similar information about spatial motion. Table 3 shows that DIR is superior to RIR in calculating 3D distances to the COM (*P = *.000). The superiority of DIR is attributable to the deformation of the breast, as well as the lumpectomy cavity caused by respiration and the effects of gravity during intrafraction irradiation; RIR has limitations in registering nonrigid tissue. Consequently, 3D distances to the COM based on DIR include centroid movements induced by respiration and the deformation of the breast. However, distances to the COM based on RIR only represent respiration-induced displacement. Therefore, DIR reflects intrafraction motion more accurately than RIR.

Many clinical studies have been performed to investigate the accuracy of DIR. Guckenberger et al^[20]^ performed DIR in adaptive radiotherapy of lung cancer to assess the dosimetric impact of anatomic changes during treatment. To evaluate dose registration accuracy, Senthi et al^[12]^ compared the spatial differences between RIR and DIR for 10 nonsmall cell lung cancer patients. They demonstrated that DIR was almost always more accurate than RIR and enabled improved the sparing of organs at risk. Our study indicates that the magnitude of the advantage of DIR is small when compared with RIR. For patients with a low local recurrence risk, APBI can achieve an adequate local control rate by targeting the location of the primary tumor and results in a shorter overall treatment time.^[21,22]^ However, multicenter randomized trials reported that APBI increased rates of adverse cosmesis and late-radiation toxicity compared with standard whole-breast irradiation.^[23,24]^ The significantly higher volume of tissue irradiated in patients with poor cosmesis is one of the potential factors explaining the increase in toxicity observed in the APBI arm of this trials. Therefore, an essential prerequisite for APBI is accurate delineation of the TB. Wang et al^[25]^ reported that although the target movement was small during free breathing, the dose variation for the ipsilateral lung was significant. The results of our study can help guide patient-specific planning target volume (PTV) construction and reduce damage to normal tissues, resulting in fewer local recurrences, minimal toxicity, and excellent cosmetic outcomes.

## 5 Conclusion

DIR can improve the overlap for GTV registration from CT_0_ to CT_50_ images from 4DCT scans. Furthermore, DIR is superior to RIR in reflecting the displacement of GTV and selected clips in the anterior–posterior direction induced by respiratory movements.
